# From Itch to Injury: An Emerging Link Between Allergic Conjunctivitis, Eye Rubbing and Lid Wiper Epitheliopathy

**DOI:** 10.3390/metabo16100779

**Published:** 2026-10-14

**Authors:** Luke X. Chong, Olivia Jagiello, Rinu Thomas, Ereeny Mikhail, Serap Azizoğlu, Moneisha Gokhale

**Affiliations:** 1 School of Medicine (Optometry), Faculty of Health, Deakin University, Waurn Ponds, Geelong, VIC 3216, Australia; luke.chong@deakin.edu.au (L.X.C.); s224179485@deakin.edu.au (O.J.); s223602085@deakin.edu.au (R.T.); e.mikhail@deakin.edu.au (E.M.); serap.azizoglu@deakin.edu.au (S.A.); 2 NeuroAllergy Research Laboratory (NARL), School of Life and Environmental Sciences, Deakin University, Waurn Ponds, Geelong, VIC 3216, Australia; 3 Institute for Mental and Physical Health and Clinical Translation (IMPACT), Deakin University, Geelong, VIC 3216, Australia

**Keywords:** allergy, allergic conjunctivitis, ocular allergy, ocular itch, lid wiper epitheliopathy, eye rubbing, dry eye, multi-omics

## Abstract

Allergic conjunctivitis (AC) is a common inflammatory ocular surface disease characterised by ocular itch, conjunctival redness, tearing, and frequent eye rubbing. Although the link between AC, eye rubbing and development of keratoconus is well established, the relationship between AC and lid wiper epitheliopathy (LWE) is less understood. Recent studies have identified a potential association between AC and LWE, suggesting that friction-induced damage to the lid wiper region may represent an under-recognised consequence of allergic conjunctivitis. This review synthesises current evidence linking allergic conjunctivitis, eye rubbing, ocular surface friction, and LWE, while highlighting emerging mechanistic insights from multi-omics research. LWE has potential as a novel clinical indicator of AC and may provide additional diagnostic value alongside other established biomarkers.

## 1. Introduction

Ocular allergy (OA) is defined as a collection of immunoglobulin E (IgE) and non-IgE-mediated ocular hypersensitivities to harmless environmental allergens, such as dust or pollen [1]. It is estimated to affect 15–20% of the global population [2] and is often a component of allergic rhinitis (also known as hay fever), a condition where almost one in four Australians (23.9%) are affected [3]. IgE-mediated allergic conjunctivitis (AC) is the most common subtype of OA [4]. Symptoms of AC include ocular redness, irritation, tearing, conjunctival and lid oedema, and the pathognomonic symptom of ocular pruritus (commonly known as itchy eyes) [1,5,6]. 

Ocular itch in AC arises after allergen sensitisation, which leads to the production of IgE by adaptive immune cells [1]. The IgE antibodies detect and cross-link with allergens to initiate pro-inflammatory degranulation of innate immune mast cells [1]. Among these pro-inflammatory mediators is the pruritogen histamine, which binds to histamine receptors H1 and H4 on sensory nerves to initiate a central sensation of itch, and by extension, eye rubbing [7]. Given the growing prevalence of AC, its significant quality-of-life impacts as well as its role in keratoconus pathogenesis and myopia progression, increasing research is being dedicated to identifying new potential diagnostic signs of OA [8,9]. One such emerging indicator is lid wiper epitheliopathy (LWE) [2,5,10,11]. 

**Figure 1:**
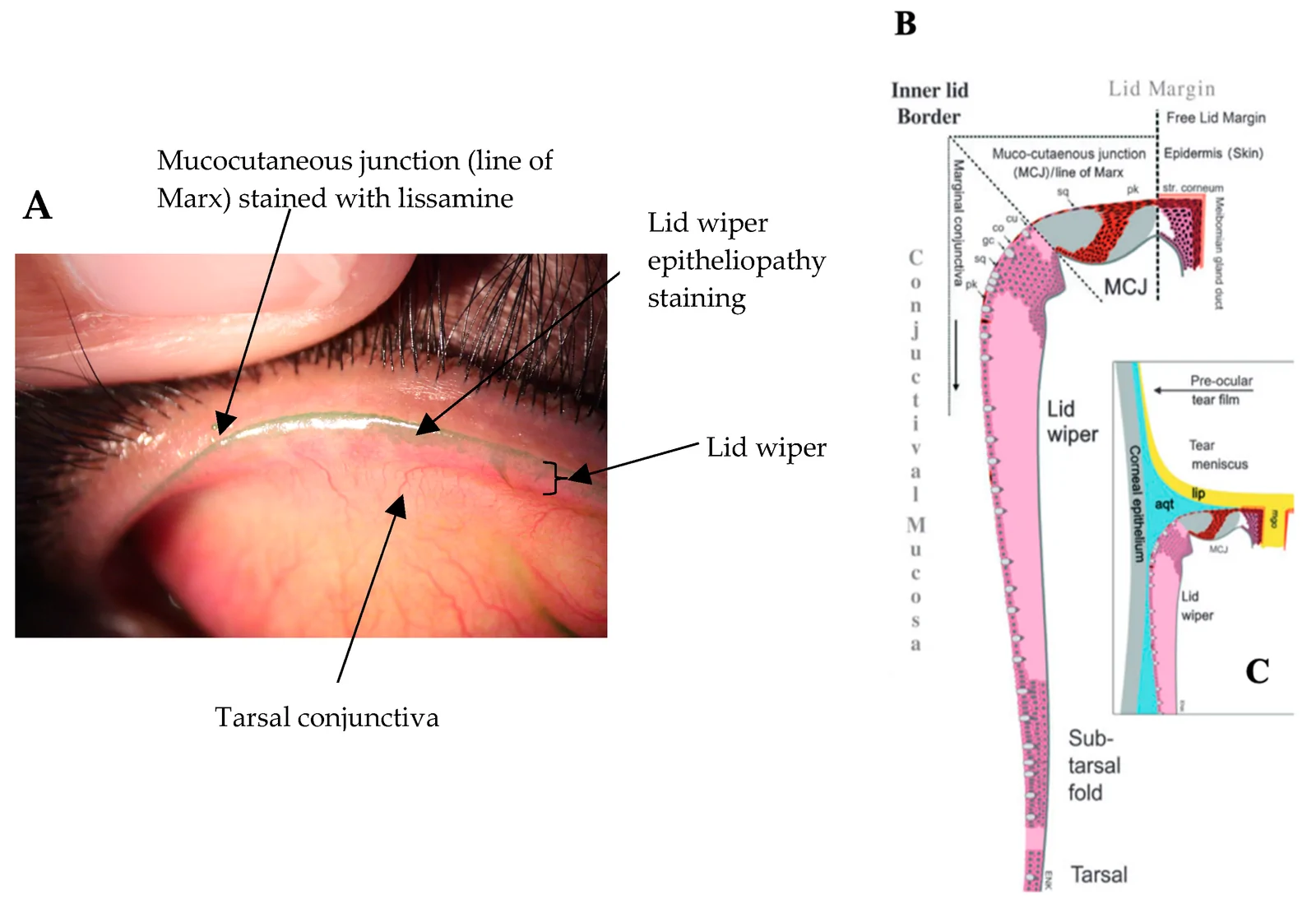
(**A**) Anatomical location of the lid wiper, seen as a 100-micron rise of epithelial tissue on the posterior-distal aspect of an everted upper lid. (**B**) Diagram of the lid margin showing the transition from para-keratinised cells of the mucocutaneous junction to atypical cuboidal and columnar epithelia interspersed with goblet cells at the lid wiper. (**C**) Diagram illustrating the relation of the lid wiper to the cornea and to the pre-ocular tear film. Figures (**B**) and (**C**) are reproduced from Knop et al. (2011) [12] with permission of WILEY via Copyright Clearance Center.

The lid wiper is a region of elevated epithelial tissue located on the posterior-distal aspect of the upper and lower palpebral conjunctiva [12,13,14]. Histologically, it is distinct from conjunctival tissue, being comprised of cuboidal epithelia interspersed with mucin-producing goblet cells and increasingly para-keratinised epithelia as the tissue approaches the distal mucocutaneous junction, clinically referred to as the line of Marx (Figure 1A) [12,15]. The structure represents the primary point of contact between the lids and the globe, facilitating the distribution of the tear film across the ocular surface during blinking to create a smooth air-tear interface and promote optical clarity [12,14,16]. The tear film occupies the interface between the lid wiper and the globe, where it functions as a biophysical lubricant, reducing friction and enabling smooth blinking. This lubricating property is conferred by its complex trilaminar structure, composed of a superficial meibomian-expressed lipid phase, a dynamic mucin-aqueous gel phase and a deep glycocalyx comprised of epithelial membrane-associated mucins (MAMs) [17]. Disruption to tear film homeostasis, known as tear film instability, predisposes the ocular surface, including the lid wiper, to frictional stress and ocular inflammation [18].

LWE refers to the damage of the delicate epithelial tissue of the lid wiper and is thought to arise from increased friction between the lid wiper and the globe [14,19]. Clinically, this damage is observed as staining of the upper or lower lid wiper on instillation of vital dyes, such as sodium fluorescein and lissamine green (Figure 1A), and is associated with ocular discomfort, dryness and irritation [19,20]. LWE is a common association with high ocular surface friction states, including contact lens wear and dry eye disease (DED) [20,21]. It is estimated that approximately 88% of symptomatic DED patients have some level of LWE (*p* < 0.0001) [22], with the Tear Film and Ocular Surface Society Dry Eye Workshop III (TFOS DEWS III) considering LWE as a diagnostic indicator of DED [23]. However, a recent discovery of an association between lower LWE and AC has emerged, which was suggested to be attributed to increased friction resulting from itch-induced eye rubbing in AC [11].

In this review, the primary intended clinical population is patients with AC, including seasonal and perennial forms. Vernal keratoconjunctivitis (VKC) and atopic keratoconjunctivitis (AKC) are severe, chronic forms of allergic ocular disease with mixed IgE-mediated and inflammatory mechanisms, and are not the target population. VKC and AKC evidence is cited only where AC-specific data are lacking and is identified as such. DED and keratoconus are treated as reference conditions, as DED provides an important clinical context in which the relationship between tear film instability, lid wiper friction and LWE has been studied, and keratoconus as an important ocular condition in which eye rubbing forces have been quantified. Although not the primary target population, findings from these groups are used to inform relevant mechanisms and associations but are not considered equivalent to evidence in AC. Where evidence is extrapolated from these reference populations to AC or LWE, this will be explicitly acknowledged.

This literature review aims to synthesise current knowledge of behavioural eye rubbing, ocular surface friction, multi-omics, and LWE to clarify their mechanistic links and highlight gaps in the understanding between LWE and AC.

## 2. Methods

In this critical narrative review, a targeted literature search was conducted using PubMed and Web of Science databases. Literature published between January 2016 and September 2026 were prioritised, although earlier studies were also included in instances where they provided seminal evidence. The search was last updated on 28 September 2026. 

Search terms that were used (both in isolation as well as in combination with Boolean operators) included “lid wiper epitheliopathy”, “eye rubbing”, “ocular allergy”, “ocular itch”, “allergic conjunctivitis”, allergic rhinitis”, “dry eye disease”, “keratoconus”, “ocular surface friction”, “tear film mucins”, “tear film biomarkers”, “tear film instability”, “metabolomics”, “proteomics”, “lipidomics”, and “multi-omics”. 

Selection was based on relevance to the review with an emphasis on studies that addressed the proposed underlying mechanism and clinical relationships between these concepts, and articles were excluded if they were either not published in English, were conference abstracts without accompanying full text, or did not provide primary evidence relevant to the mechanistic or clinical associations addressed in this review. Reference lists of retrieved articles were also manually searched for additional relevant studies.

## 3. Eye-Rubbing Patterns and Behaviours

In AC, ocular itch drives frequent and sometimes forceful eye rubbing. Eye rubbing involves the manual exertion of mechanical pressure onto the globe and ocular adnexa, typically through a closed eyelid in response to an irritating or pruritogenic stimulus [5,24]. In a cross-sectional prevalence study, Stull et al. (2017) found that chronic ocular itch was present in 29.5% (*n* = 118) of their general study population (*n* = 400) and was associated with ocular surface diseases (OSDs) such as DED, blepharitis, and AC [10]. Indeed, the prevalence of chronic ocular itch was greatest among the AC subpopulation (*n* = 86) of this study, with 51.8% (*n* = 45, *p* < 0.001; OR = 5.0) experiencing sensory and behavioural ocular itch [10]. Hage et al. (2023), who examined a similar panel of OSD and keratoconus study participants (*n* = 153), found that ocular itch was present in 57.6% (*n* = 72, *p* < 0.0001) of their eye-rubbing subpopulation, and that 15 of 16 AC subjects rubbed their eyes [5]. It is reasonable then to presume that ocular itch similarly drove the high prevalence of eye rubbing observed by Thomas et al. (2025), who found that 87.5% (*n* = 14) of their 16 allergic rhinitis participants engaged in eye rubbing, while no healthy control subjects did [11]. Together, these findings establish ocular itch as a principal trigger for eye rubbing in AC, prompting further exploration of the frequency and duration of eye rubbing episodes. 

Quantifying ocular itch and eye rubbing location, frequency and duration in AC provides insight into the individual risk of ocular surface and tissue damage. Thomas et al. (2025) characterised eye rubbing frequency in their allergic rhinitis subpopulation, with 50% reporting occasional rubbing, 25% often and 12.5% more often, with only 2 participants reporting none [11]. Hage et al. (2023) further demonstrated that despite the well-described association between keratoconus and behavioural eye rubbing, daily eye rubbing prevalence was greater in the OSD group than the keratoconus group (55.6% vs. 32.3%; *p* = 0.009) [5,25,26]. A modified symptom assessment in dry eye (SANDE) score corroborated this, with rubbing frequency scoring higher in the OSD group (frequency 63.5% vs. 44.4%, *p* ≤ 0.001) [5]. Given the self-reporting nature of these data, however, true rubbing frequency may be underestimated, as participants are prone to under-report habitual behaviours [27]. 

Regarding episode duration, Stull et al. (2017) reported that mean duration of a single episode of chronic itch lasted 4 h (range = 2 s to 7 days) with a mean intensity of 5.6 ± 2.6 (mean ± standard deviation) on a 10-point scale [10]. Notably, however, this sample included multiple ocular conditions and was not limited to AC, and only reported duration of ocular itch episodes and not eye rubbing specifically. On the other hand, Thomas et al. (2025) found that of the participants who engaged in eye rubbing, most (64.3%) reported that eye rubbing episodes lasted only a few seconds [11]. 

In terms of location, there is an apparent lack of agreement amongst current literature. Stull et al. (2017) discovered that the most commonly reported locations of chronic ocular itch were the inner canthi (20.3% of total study sample), lower eyelids (20.3%) and upper eyelids (19.5%) [10]. Conversely, Thomas et al. (2025) found that 50% of participants with hay fever in their study usually concentrated on the outer canthi when eye rubbing [11]. Nonetheless, across these studies, eye rubbing emerges consistently as a high-frequency behaviour driven by inflammatory ocular itch, underscoring the importance of characterising, not only by its temporal patterns, but also by the mechanical force it imparts on the ocular tissues. 

Behavioural eye rubbing techniques and force is an area of active research for its role in keratoconus pathogenesis. In allergic eye disease, Thomas et al. (2025) characterised eye rubbing intensity by self-report in their allergic rhinitis population, with 50% of their study sample citing use of a soft technique, 42.9% firm and 7.1% forceful [11]. Hage et al. (2023), using the SANDE score, recorded a mean eye rubbing intensity of 58.90/100 in their OSD group versus 42.50/100 in their keratoconus group (*p* ≤ 0.001) [5]. Hafezi et al. (2020) however, sought to quantify rubbing force more directly, by having keratoconus subjects (*n* = 57) apply their preferred eye rubbing method (i.e., fingertip, fingernail or knuckle eye rubbing) onto a precision scale [24]. Fingertip rubbing was the most common method (51%, *n* = 29), exerting an average of 4.3 ± 4.0 Newtons (N), while knuckle rubbing (44%, *n* = 25) generated the greatest force at 9.6 ± 4.0 N. However, it should be noted that Hafezi et al. (2020) [24] did not report the contact area involved with each rubbing technique, and as pressure is a function of both the force that is applied as well as the contact area over which it is distributed (Pressure = Force/Area), it is not possible to directly compare their results with the normal superior eyelid pressure of 8.0 ± 3.4 mmHg reported by Shaw et al. (2010) [28]. Despite this limitation, even conservative estimates of fingertip and knuckle contact area place the resulting pressures from eye rubbing far above typical baseline eyelid pressure, supporting the suggestion that eye rubbing generates substantial mechanical load and risk to the ocular surface. For example, if we assume a broad contact area of 2 cm^2^, calculated pressures for fingertip and knuckle rubbing would equate to 161.27 mmHg and 360.03 mmHg respectively. While the method implemented by Hafezi et al. (2020) [24] cannot fully replicate in vivo tissue deformation or simulate eye rubbing motions, these forces substantially exceed reported baseline upper eyelid pressures.

Collectively, these data suggest that eye rubbing in AC is not only frequent but potentially forceful, with significant implications for cumulative mechanical load. As with temporal parameters, however, intensity estimates derived from self-reporting may also be susceptible to under-reporting bias [27]. A prominent gap emerging from this evidence is the effect of eye rubbing on ocular surface friction parameters, with most literature focusing on the role of eye rubbing as a mechanical load in keratoconus [8,24,26,29]. An ocular tribometer to measure ocular surface friction in vivo is currently in development and shows promise for use in OSD, including AC [30]. This tool represents a promising avenue for future research, characterising how different eye rubbing techniques alter ocular surface friction parameters which is of relevance given the association between AC and LWE [11]. Ultimately, future research should clarify the behavioural and mechanical pathways through which repeated and forceful eye rubbing contributes to cumulative friction ocular surface damage in AC [5,11,24,30].

## 4. Frictional Forces and Lid Wiper Histology

Friction is defined as the force that resists the movement of articulating surfaces and is influenced by movement speed, mechanical load and fluid lubrication [6,31]. The significance of these parameters becomes apparent when considering the unique histology of the lid wiper. Tissues exposed to repeated friction are conventionally comprised of stratified squamous epithelium, providing an abrasion-resistant cytoarchitecture [14,32]. The lid wiper, however, deviates from this convention as histological studies have identified 8-12 strata of cuboidal and columnar epithelia lining the region (Figure 1B) [12,15]. This more delicate and softer cell structure is thought to minimise mechanical damage to the opposing corneal and globe surfaces on blink, but may confer a heightened dependence on tear film lubrication for friction resistance and highlight a vulnerability in states of tear film instability [12,15].

**Figure 2:**
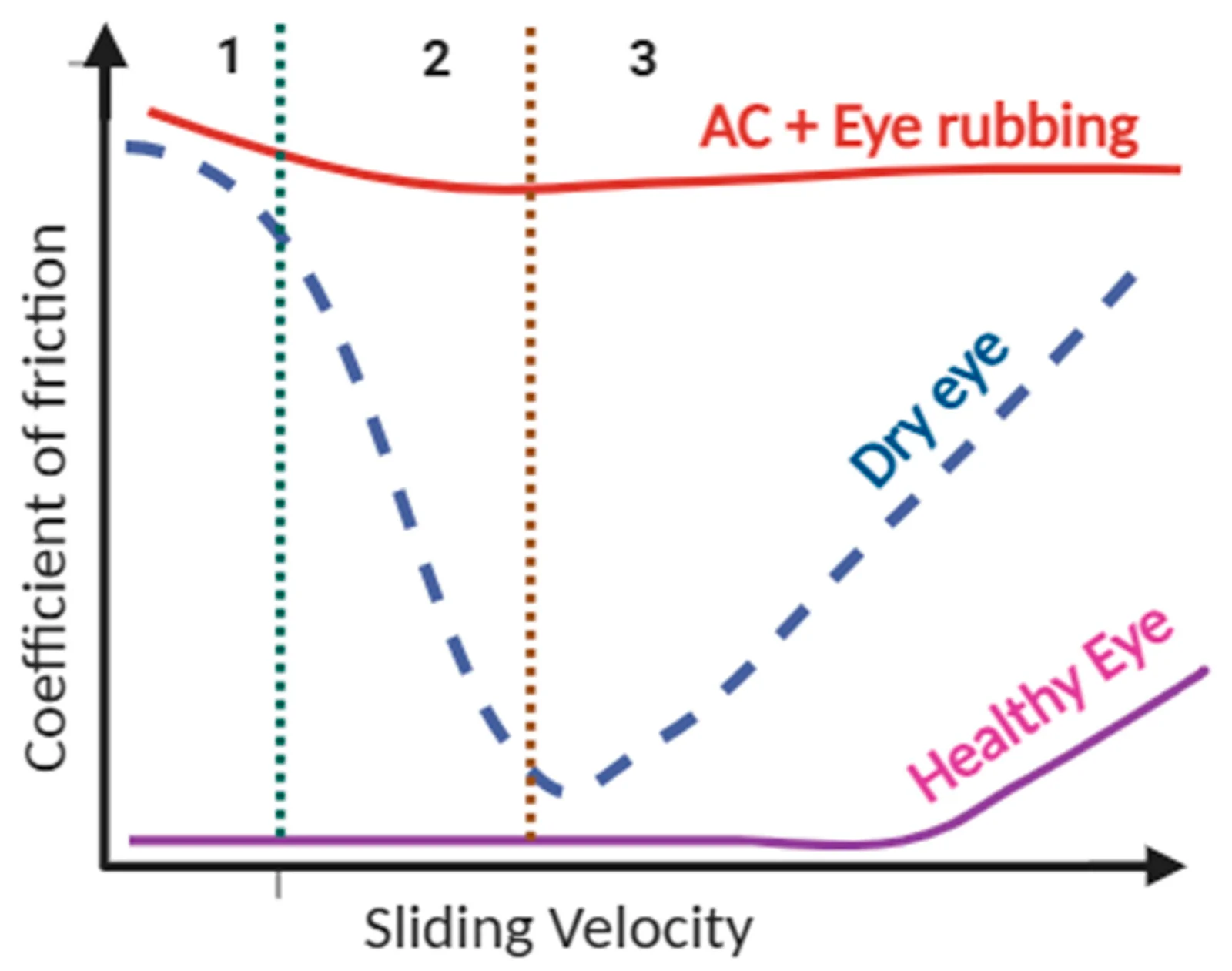
Schematic Stribeck curve illustration depicting the theoretical coefficient of friction as a function of sliding velocity in healthy individuals and those with dry eye, as well as the predicted hypothetical curve for the combination of allergic conjunctivitis with eye rubbing. The coefficient of friction is very low in normal eyes with thicker tear films. It follows the classic Stribeck curve in the case of dry eye as proposed by Pult et al. [18] and the coefficient of friction will be theoretically higher in the case of ocular allergy associated with eye rubbing. 1 = boundary lubrication, 2 = mixed lubrication, 3 = hydrodynamic lubrication. Figure constructed by Rinu Thomas using BioRender.

The healthy ocular surface exists in a state of relatively low friction due to an intact and functional tear film that minimises direct tissue interaction between the lid wiper and the globe. Under physiological conditions, blinking occurs without the addition of significant mechanical pressure such that tear film lubrication is maintained across a range of surface-sliding velocities [6,18]. The Stribeck curve is a widely accepted model of friction (Figure 2). This model is used to understand ocular surface lubrication. It describes increased friction during low- and high-velocity tissue interactions, called the boundary and hydrodynamic phases, respectively, which arise during the corresponding slow and fast stages of blink [6,18]. During the boundary phase, friction increases through greater surface contact between sliding tissues. The mixed phase represents a phase of low friction during the transition from boundary to hydrodynamic friction. The hydrodynamic phase represents slight separation between surfaces, meaning friction arises from shear force within the medium between the articulating surfaces. 

A graphical theoretical depiction of a Stribeck curve comparing ocular surface friction between healthy individuals, those with DED and AC with associated eye rubbing, is shown in Figure 2. It should be noted that Figure 2 illustrates a conceptual hypothesis for AC with eye rubbing, and that ocular friction has not been directly quantified in AC to date. In DED, the mucin layer and glycocalyx brushes are damaged, which results in increased friction at low velocity due to tear insufficiency [33]. There is evidence of increased tear film viscosity due to altered tear film properties in dry eye that lead to an increase in friction at high velocity. On the other hand, in AC there is limited research in this area regarding viscosity; however, there is evidence that the mucins and glycocalyx are altered in atopic keratoconjunctivitis and vernal keratoconjunctivitis [34]. Additionally, tear film volume and flow are significantly reduced in AC [35,36]. This has the potential to damage the sliding partners, and this effect is exacerbated further with eye rubbing. Theoretically, in the context of AC, friction can be induced due to two mechanisms: spontaneous blinking and eye rubbing due to ocular itch. Additionally, the abnormal tear film in AC makes the ocular surface more susceptible to damage due to friction. This might exceed the tolerance level of corneal and lid wiper sensitivity, thus leading to manifestation of patient symptoms [18]. 

In a healthy eye, friction at low sliding velocities is mitigated by the glycocalyx, primarily comprised of glycosylated membrane-associated mucins MUC1, MUC4 and MUC16 [6,37,38,39]. This mucin structure retains aqueous and lipid tears on the ocular surface while forming a polymer brush minimising the boundary friction that elsewise arises through direct contact between the lid wiper and the globe [6,38,39]. During the hydrodynamic phase, by contrast, friction arises from viscous resistance to shear forces within the fluid interfacing the sliding surfaces [6,40]. Here, secreted mucins, principally MUC5AC, bind with aqueous tears to form a viscoelastic hydrogel critical to lubrication during the hydrodynamic phase of lid movement [6,40,41]. Under the shear stress generated by lid movement, this hydrogel undergoes shear thinning whereby its viscosity decreases proportionally to the shear rate, effectively reducing fluid resistance and hydrodynamic friction [6,40]. Lipid tears contribute to this system more indirectly, through preservation of tear film morphology and prevention of aqueous evaporation [18,42,43]. Nonetheless, tear film integrity of both the lipid and mucin layers are essential to ocular surface lubrication across the full range of friction states outlined by the Stribeck curve. Therefore, compromise of this system disrupts lubrication and increases friction at both the boundary and hydrodynamic phases which is thought to be the principal aetiological driver of LWE in OSD, including DED and potentially AC [18,42,43].

This makes the finding of a unique goblet cell population in the lid wiper highly intriguing [15]. Secreted mucin is produced by modified epithelial goblet cells; Knop et al. (2012), in a histological examination, found a rich deposit of goblet cells within the lid-wiper but observed that a significant proportion of these did not stain positive for mucin MUC5AC [15]. MUC5AC was thought to be richly and ubiquitously expressed throughout the human conjunctiva; however, this understanding was based on samples taken exclusively from the bulbar and not palpebral conjunctiva [41,44]. As such, these findings suggest a unique mucin composition local to the lid wiper or increasing reliance on glycocalyx mucins [15]. Choi and Tichenor (2024) also characterised the MAM profile of the upper lid and lid wiper, and though no significant difference in MAM expression was found between DED and healthy states, they found that MUC1, 4 and 16 were each present within the upper lid wiper, which supports the notion of MAM glycocalyx boundary lubrication on the ocular surface [45]. No subsequent studies have sought to characterise the secreted mucin profile within the lid wiper goblet cells, which, given the absence of MUC5AC highlights a promising area for further research to inform the lid wiper’s friction resistance or vulnerability in OSD. 

However, a major caveat in the studies by Knop et al. (2012) and Knop et al. (2011) is the use of histological analysis of the lid wiper from cadavers with a mean age of 77 years, which may not be representative of other populations outside of this demographic [12,15]. Given that the incidence of DED and, presumably, LWE increases with age, further studies should seek to characterise lid wiper histology in younger populations both with and without DED to obtain a better understanding of homeostatic histology and the changes seen in LWE. Cumulatively, in conjunction with the lid wiper’s atypical epithelial histology and mucin secretion profile, this renders the lid wiper a histologically unique and potentially vulnerable structure to frictional stress; any disruption to the delicate homeostatic balance of the tear film compromises this lubrication system, increasing frictional stress during blink and eye rubbing, providing a biomechanically feasible mechanism for LWE pathogenesis.

## 5. Effect of Mechanical Pressure on Lid Wiper Epitheliopathy

Whilst it is not currently possible to measure baseline ocular surface friction in AC directly, it is plausible that friction is heightened between articulating surfaces under increased mechanical pressure [18,46]. A study by Yamamoto et al. (2016) demonstrated increased burden of lower LWE in people with increased lower lid tensile pressure on the globe as measured by blepharo-tensiometer [47]. In study participants with grade 3 lower LWE, mean lower lid pressure was 27.9 ± 2.8 mmHg as compared to 19.7 ± 1.3 mmHg in participants without LWE (*p* < 0.05) [47]. However, this study was limited by the sample being exclusively of a Japanese ethnic origin with a mean age of 28 years (range = 18–71 years). Whilst increasing age represents reduced tear quality and eyelid laxity causing reduced tensile pressure, Li et al. (2018) demonstrated that LWE prevalence and severity were positively associated with Asian ethnicity (*p* < 0.005) [48]. This highlights a gap in our understanding of lid pressure across ethnicities that might further evidence the lid pressure hypothesis in the incidence of LWE. Cumulatively, however, this evidence suggests that LWE arises when mechanical pressure and any associated frictional stress exceed the protective capacity of the tear film and lid wiper. Under these conditions, prolonged friction precipitates epithelial, goblet cell and glycocalyx distress, and thus leads to LWE. 

## 6. Lid Wiper Epitheliopathy and Its Association with Dry Eye Disease Parameters 

**Figure 3:**
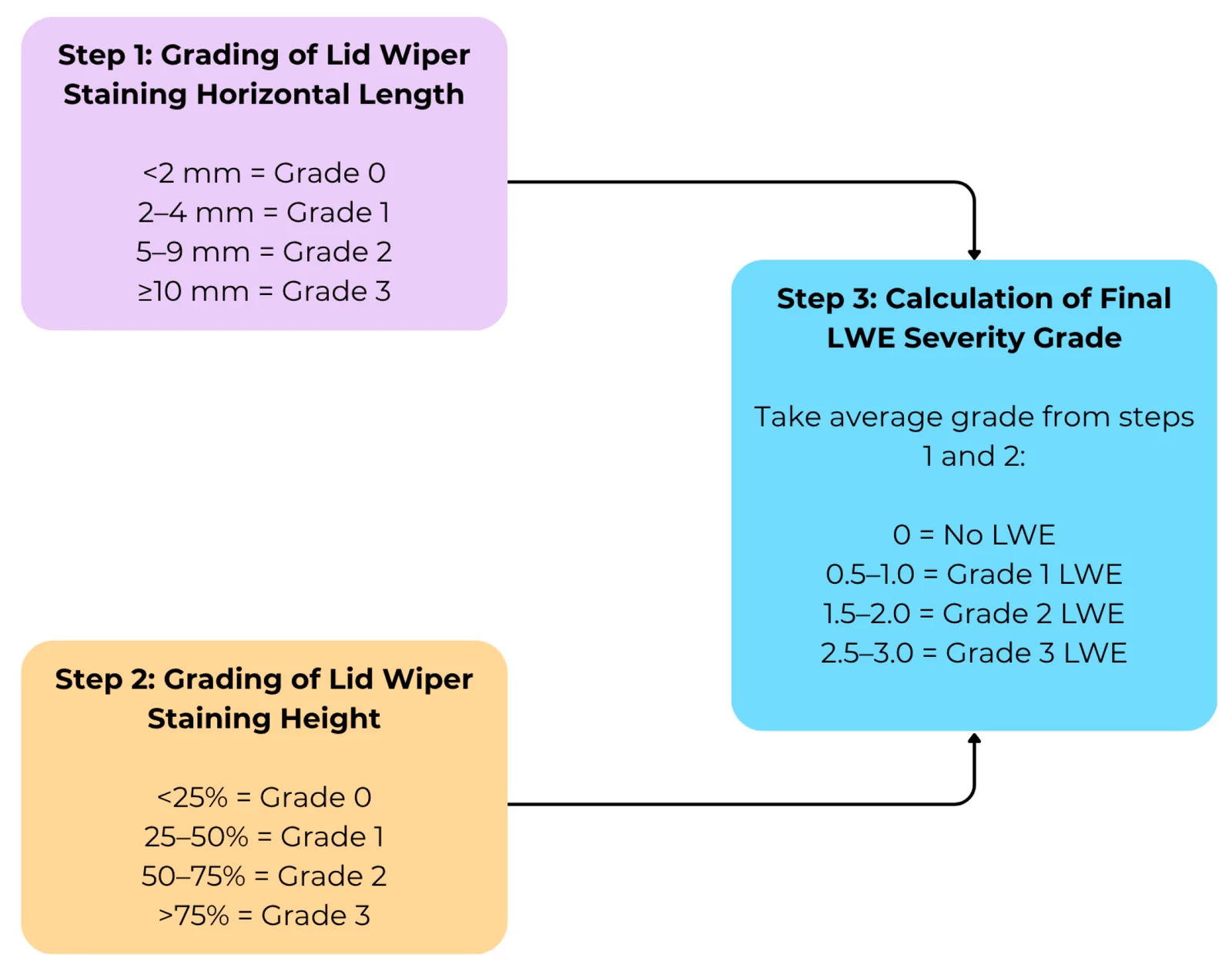
The three-step process used to grade LWE severity using the protocol developed by Korb et al. (2005) [13]. Horizontal length of staining in millimetres (step 1) and width of lid wiper staining as a percentage of total lid wiper width (step 2) are first graded separately. The average of the length and width staining grades then form a single combined LWE grade score (step 3).

Associations between LWE and parameters of tear film quality have formed the principal evidence base for the current understanding of LWE pathogenesis, given the current absence of technology to directly measure ocular surface friction [19,20,30]. In research, LWE is commonly assessed using a combination of vital dyes, namely, lissamine green and sodium fluorescein, under slit lamp biomicroscopy [47,49]. The staining is then graded semi-quantitatively according to its height (excluding the width of the mucocutaneous junction) and length forming a combined average score, as described by Korb and colleagues (Figure 3) [13]. Higher LWE grading scores reflect greater epithelial cell damage and, although not a direct measure of friction between the lid wiper and the globe, LWE is widely believed to be an indicator of increased ocular surface friction due to inadequate lubrication [13,14,21,22]. As such, the current understanding of LWE pathogenesis has emerged from its association with DED, recognised ubiquitously as a state of increased ocular surface friction from reduced tear film quality and lubrication [19,20]. 

There are several studies that correlate DED diagnostic parameters to LWE staining [19,48,49]. A study by Liu et al. (2020) explored the relationship between non-invasive tear meniscus height (TMH) and LWE severity, finding that a TMH less than the 0.2 mm diagnostic standard was associated with a higher LWE grading (LWE grading: 1.81 ± 0.41 <0.2 mm TMH vs. 1.39 ± 0.47 ≥0.2 mm TMH, *p* < 0.0001) [49]. Similarly, non-invasive tear break-up time (NITBUT), which measures tear film stability, was lower in participants with higher LWE staining scores (r = −0.7820, *p* < 0.0001) [49]. Li et al. (2018) found a similar result, with a shorter NITBUT being associated with both an increasing length and width of LWE staining (*p* < 0.005 length; *p* = 0.01 width); however, neither NITBUT nor LWE scores were disclosed [48].

A limitation of both studies by Li et al. (2018) and Liu et al. (2020), however, was the use of a subjective LWE grading approach [48,49]. Such an approach relies heavily on the accuracy and competency of the grader to make judgments about LWE severity, which often may be difficult to determine for human graders, as the lid wiper region is not well delineated. Kunnen et al. (2018) found that human examiners tended to underestimate width and overestimate the height of lid wiper staining, highlighting the possibility for inter- and intra-examiner subjectivity errors, which may have skewed findings [50]. Recent advances in LWE analysis have favoured semi-objective MATLAB image segmentation software, that maps length, height and area with a greater degree of spatial and lissamine-hue sensitivity than human examiners from slit lamp photographs, which should be employed in further research in LWE [50,51]. 

A semi-automated approach using a custom MATLAB program to automatically calculate LWE width and height resulted in greater repeatability compared to human grading [50]. Furthermore, Ravikumar et al. (2023) developed an automated Hue-Value LWE grading algorithm that quantifies staining using the Hue and Value domains of the Hue–Saturation–Value colour space after manual selection of the region of interest [51]. This approach removes the need for manual tracing of LWE staining, thus simplifying the procedure and also improving grading efficiency. Overall, repeatability between graders was high using this approach, with no statistically significant difference in inter-grader variability observed for LWE height or area (*p* > 0.05, paired *t*-test) and a statistically significant but clinically insignificant mean difference of 0.16 mm between graders for width (*p* = 0.031, paired *t*-test). Cumulatively, however, a theoretical consensus does emerge from these findings that confirms that measures of tear film instability, namely, TMH and NITBUT, are consistently and positively associated with LWE staining, supporting the notion that compromised surface lubrication is pathogenic for LWE. Furthermore, it should also be noted that neither of the studies by Li et al. (2018) or Liu et al. (2020) analysed the lower lid wiper region [48,49].

Tear mucins have emerged as essential mediators of lid wiper lubrication during boundary and hydrodynamic stages of friction, and there is a consensus that DED represents a state of glycocalyx-mucin collapse [18]. Korb et al. (2005), in their seminal work on LWE in DED, propose that loss of tear film volume, seen clinically as reduced TMH, increases reliance on the glycocalyx for lubrication at the lid wiper, thereby predisposing the tissue to friction during both boundary and hydrodynamic phases of blink [13]. Supporting this, an in vitro study that used confocal microscopy to examine the relative secretion of membrane-bound mucin MUC1 in human conjunctival epithelial cell responses after exposure to low-level shear stress (0.5 dyne/cm^2^) found a reduction in MUC1 secretion after 30 min of shear stress exposure (*p* < 0.05) [40]. This finding suggests that LWE might arise under conditions of over-reliance and damage to glycocalyx mucins at the lid wiper when frictional and shear stress exceed mucin capacity for lubrication. 

It is evident that the lubricating function of the glycocalyx depends not only on mucin abundance but also on post-translational glycosylation of MAMs [52,53]. Specifically, the addition of O-glycan groups to MAMs allows for the binding of galectin-3, forming a stabilised glycocalyx polymer brush that is essential for epithelial barrier integrity and low-friction boundary lubrication [54]. In DED, increased tear galectin-3 concentrations are believed to reflect altered mucin glycosylation [55,56], preventing normal galectin-3 binding and compromising glycocalyx architecture [54]. MUC16 undergoes the addition of an O-acetyl sialic acid and was found to be critical to glycocalyx barrier function and prevention of ocular surface staining [52,53]. This finding points to either its sparsity or loss of glycosylation in the construction of the glycocalyx. This supports the idea that functional integrity of the glycocalyx depends on appropriate mucin glycosylation rather than mucin abundance alone. Together, these findings provide a mechanistic explanation for how tear film instability in DED can lead to collapse of the glycocalyx polymer brush, increased friction and ultimately the epithelial damage characteristic of LWE.

Alterations in goblet cell density and free-mucin secretions have been observed in DED with LWE. Dou et al. (2025), on investigating these parameters, found a reduction in both goblet cell density and MUC5AC concentration in DED subjects with LWE compared to DED subjects without LWE (Goblet cell density: 91.54 ± 70.32 cells/mm^2^ vs. 121.0 ± 64.22 cells/mm^2^, *p* = 0.045; MUC5AC concentration: 44.91 ± 20.81 ng/mg vs. 61.96 ± 22.12 ng/mg, *p* = 0.050) [57]. This finding suggests that lid wiper goblet cell dysfunction and reduced mucin secretion might be either a cause or consequence of LWE, requiring further research to delineate the mechanism behind these changes as future interventional targets in DED and, possibly, AC. Current evidence does suggest that epithelial cells (of which goblet cells are a modification) are prone to attrition under mechanical stress, suggesting that biophysical stress as friction or possibly eye rubbing might be precipitant of epithelial and goblet cell damage, and thus LWE [58]. To further support this assertion, Kase et al. (2017) demonstrated an increase in lid wiper goblet cell count after treatment with topical rebamipide (a mucin secretagogue drug) relative to the control group (29.4 ± 13.8 vs. 12.2 ± 9.4; *p* = 0.0367) [59]. Subsequently, a case study (*n* = 2) determined that a three-week course of topical ophthalmic rebamipide was able to resolve LWE staining [60]. Cumulatively, these findings support the notion that LWE arises secondary to mucin loss, evidenced both by the association between LWE and reduced goblet cell density and the improvement in LWE after mucin-targeted therapy. Further research should characterise goblet cell-derived mucins and transmembrane mucins at the lid wiper, not only to better define their role in friction resistance but to identify potential therapeutic targets for LWE.

## 7. Allergic Conjunctivitis: Similarities to Dry Eye Disease and Increased Susceptibility to Lid Wiper Epitheliopathy

As with DED, AC has also been reported to be associated with tear film instability [61,62]. Tear volume, estimated as TMH, is reduced within these groups, as studies show an average TMH of 0.199 ± 0.099 mm in DED participants [63]. Similarly, TMH was reduced in another study on AC participants, demonstrating a TMH of 0.26 ± 0.10 mm compared to the 0.44 ± 0.08 mm control group (*p* < 0.0001) [64]. These findings demonstrate the similarity in tear volume loss profiles between AC and DED. Though more pronounced in DED, the statistically significant reduction in tear volume in AC might mechanistically rationalise the significant symptomatic and clinical overlap between DED and AC, and relate to pathogenesis of LWE; however, tear stability is also an important consideration [61,62]. 

NITBUT and fluorescein tear break-up time (FBUT) are often used to assess tear stability. In AC, FBUT findings across several studies, detailed in Table 1, are consistent in showing reduced tear film stability in AC compared to healthy controls [64,65,66,67]. Significant variability exists within this data, however, ranging between a mean FBUT of 3.9 and 15.7 seconds [65,67]. Methodological differences may explain these discordant findings, whereby sodium fluorescein was used by Polat and Tuncer (2014) [66], topical anaesthesia was administered by Dogru et al. (2015) in a paediatric population [65], and Suzuki et al. (2006) used fluorescein dye solution [67], which likely differentially affected tear stability and inflated tear volume [68]. However, Wei et al. (2024) utilised NITBUT [64], which preserves organic tear composition and volume. Despite this advantage, another study by Potenza et al. (2026) demonstrated surprisingly low inter-ocular concordance with NITBUT (Pearson’s r = 0.38), which was not seen for FBUT (Pearson’s r = 0.67) [69]. This collectively suggests that tear film instability in AC is variable between populations but consistently lower than healthy controls across testing modalities. Further, Potenza et al. (2026) in a cross-sectional study demonstrated a mean NITBUT of DED subjects (*n* = 295) was 5.04 ± 3.33 s and 4.99 ± 3.13 s, and FBUT of 4.95 ± 2.84 s and 5.17 ± 2.08 s in right and left eyes, respectively [69]. This demonstrates that AC represents a similar, albeit less severe, state of tear film instability than DED.

To further evidence this hypothesis, studies have demonstrated that conjunctival goblet cell count is reduced in AC. In a murine model of AC, the population of filled goblet cells interspersed within conjunctival epithelia on whole-mount was significantly reduced for 0–24 h after sensitised exposure to allergens, with peak loss at 6 h (*p* < 0.001) [70]. This study also demonstrated that MUC5AC gene expression was diminished immediately after allergen exposure (*p* < 0.05); however, it rebounded after 24 h. These findings lend credit to the understanding that the mucin-aqueous hydrogel is essential to tear film stability, as studies have demonstrated that tear film mucin content is reduced in participants with DED, though discussion around the relative reduction in MUC5AC concentration (nanograms/milligram (ng/mg)) differs with Uchino et al. (2014), showing MUC5AC is diminished in participants with DED compared to healthy controls (3.5 ± 3.2 ng/mg vs. 8.2 ± 10.4 ng/mg, *p* = 0.02) [71]. Meanwhile Duan et al. (2023) showed no statistically significant difference (*p* = 0.748) [72]. Further research should seek to clarify this discordance and investigate tear film mucin composition in AC, especially considering findings by Dou et al. (2025), demonstrating reduced MUC5AC secretion in LWE [57]. Despite this, the common reduction in tear film mucin content in both DED and AC suggests a mucin-related mechanistic similarity that likewise might predispose AC patients to ocular surface dryness, loss of lubrication and LWE.

Finally, in relating this to eye rubbing in AC, eye rubbing is established as a cause of significant mechanical pressure on the ocular surface [24]. This demonstrated the importance of the findings by McMonnies et al. (2010), who show that 15 s of moderate intensity eye rubbing caused transient (<30 min) deformation and thinning of corneal epithelial cells immediately (*p* < 0.006) and at 15 min after eye rubbing (*p* < 0.0014) [73]. This study proposed epithelial cell deformation and putative cell loss from membrane rupture as mechanisms for these results and supports the notion that eye rubbing is causative of significant ocular surface and epithelial damage. Given Knop et al. (2011) demonstrated atypical cuboidal and columnar cytoarchitecture at the lid wiper, this finding suggests that eye rubbing might exacerbate the lid wiper’s innate vulnerability to mechanical loading and frictional stress in LWE pathogenesis [12]. Further, in understanding that goblet cells are a modified mucin-producing epithelial cell, it is plausible, though not yet evidenced, that goblet cells at the lid wiper may also be lost under mechanical pressure or frictional stress [58]. This consideration is especially important in considering the recent work of Dou et al. (2025), who demonstrated reduced mucin production and goblet cell density in LWE [57]. Given the frequency and intensity of eye rubbing in AC, understanding these mechanical associations is critical [57]. To date, no studies have directly examined eye rubbing and LWE, highlighting a profound and emerging literature gap of significant mechanistic merit alongside diagnostic and therapeutic utility in AC. 

## 8. Multi-Omics Biomarkers Linking Allergic Conjunctivitis and Eye Rubbing to Lid Wiper Epitheliopathy

In recent years, biomarker-based research has gained prominence due to its ability to provide accurate and objective assessment of disease processes [74]. Biomarkers serve as measurable indicators of normal biological processes, pathogenic mechanisms, or responses to therapeutic interventions. Their identification spans multiple platforms, including clinical evaluation as well as omics-based approaches such as proteomics, lipidomics, transcriptomics, metabolomics, epigenomics, and genomics. In ocular surface research, biological samples such as tears [75,76,77,78,79,80,81,82], saliva [76,83,84,85,86,87], conjunctival cells [80,88,89], and nasal mucus [90,91,92,93,94] have been widely utilised for biomarker discovery.

It is well understood that recurrent mechanical trauma, particularly from eye rubbing associated with AC, contributes significantly to ocular surface damage [95,96,97,98]. This mechanical stress can induce the upregulation of proteins involved in inflammation, extracellular matrix remodelling, wound healing, and angiogenesis. Such molecular alterations have been well documented in both AC and keratoconus [25,77,99,100,101,102,103,104,105]. 

Recent tear proteomic studies have identified the upregulation of several differentially expressed proteins in AC compared to healthy controls, including alpha-amylase (AMY1A/B/C), Keratin 76 (KRT76), Fibromodulin (FMOD), and Dickkopf-related protein 4 (DKK4), and the downregulation of other key proteins such as Kininogen-1 (KNG1), Moesin (MSN), Septin-2, filaggrin (FLG), Keratin 80 (KRT80) and F-actin capping protein subunit alpha (CAPZA 1/2) [75,100,106,107]. These proteins are associated with epithelial remodelling, cytoskeletal reorganisation, and wound repair processes, reflecting structural alterations in the corneal, conjunctival, and lid wiper epithelia. 

Both FMOD and KRT76 are thought to be associated with allergy-induced itch and an increase in the wound healing response, structural protein expression and angiogenesis because of eye rubbing [75]. It can therefore be inferred that, as LWE worsens from increasing friction, FMOD and KRT76 are upregulated to promote wound healing and angiogenesis pathways to repair the epithelial damage on the ocular surface. However, because the underlying AC and ocular itch continue to persist, ongoing eye rubbing disrupts this repair cycle; therefore, it could be theorised that individuals with AC could be caught in this possible pathological wound-healing loop where the lid wiper is perpetually injured, partially repaired and re-injured. However, further multi-omics studies directly investigating lid wiper tissue in the presence of eye rubbing would be required to confirm this possible mechanism. 

The downregulation of MSN and CAPZA 1/2, which are integral in maintaining cytoskeletal stability and epithelial cellular integrity, further supports the link between eye rubbing and AC [75,106]; we hypothesise that the repetitive mechanical blinking over an inflamed surface in addition to the severe mechanical stress induced by eye rubbing would cause these weakened cellular structural bonds to fail, leading to the epithelial damage seen in LWE. Notably, the downregulation of KRT80, a protein critical for epithelial differentiation and structural integrity, suggests compromised barrier function at mechanically stressed sites [100]. Similarly, impairment of FLG, a key protein involved in epithelial barrier maintenance, further indicates disruption of the lid wiper region [100].

In addition to proteomic changes, micro-ribonucleic acid (miRNA) profiling has revealed the downregulation of miR-143-3p and dysregulation of miR-205-5p, miR-126-3p, and miR-516b-5p [81]. These miRNAs are implicated in pathways related to inflammation, cellular repair, and angiogenesis, supporting the hypothesis of sustained epithelial injury and repair mechanisms across the cornea, conjunctiva, and lid wiper in AC associated with eye rubbing [108,109,110]. Similarly, it has previously been discovered that the downregulation of miR-133b may be a possible predictive biomarker for allergic inflammation [111,112,113].

Lipidomic pathway analyses further support these findings, demonstrating the downregulation of choline-derived lipids, phosphatidylcholine and phosphatidylethanolamine, which result in reduced tear film stability and increased rate of evaporation from the ocular surface, as well as an altered lipid layer composition contributing to further inflammation [114,115]. This tear film instability and allergen exposure together initiate a self-perpetuating inflammatory cascade on the ocular surface. This mechanical stress activates epithelial pattern recognition receptors, triggering the release of pro-inflammatory cytokines IL-1β and IL-18 [116]. This primary insult is amplified through secondary release of IL-17A [117] and IFN-γ [118], which collectively drive epithelial dysfunction characterised by goblet cell apoptosis, mucin deficiency, and compromised barrier integrity [82]. Without a stable lipid layer to adequately lubricate the ocular surface, the coefficient of friction rises significantly [18]. Prolonged tear film instability along with cytokine storm then contribute to an increase in the likelihood of LWE to occur, recur, and progress, hence contributing to sustained chronicity of the disease.

Functional enrichment analysis of upregulated metabolites in OA suggested activation of pathways such as phosphatase and tensin homolog (PTEN) signalling and global genome nucleotide excision repair (GG-NER) [114], both of which are associated with cellular responses to oxidative deoxyribonucleic acid (DNA) damage in epithelial tissues. This may indicate ongoing DNA repair and tissue-level healing processes at the ocular surface, and could reflect an adaptive increase in wound healing responses, potentially facilitating enhanced migration of epithelial cells to sites affected by allergic itch and repetitive eye rubbing [114]. Concurrently, a significant decrease in key metabolites, including homogentisic acid, hexanoylglycine, and ornithine, was observed in the AC group compared to healthy controls. This reduction in lipid- and metabolite-associated components suggests compromised tear film stability, likely leading to increased tear film evaporation and thinning of the tear layer. Such alterations can enhance frictional forces between the lid wiper and the ocular surface during blinking, thereby exacerbating mechanical stress and epithelial damage. Key biomarkers integrated into our proposed AC and LWE mechanistic model are summarised in Table 2.

Although metabolomic evidence remains comparatively limited, it is increasingly recognised that metabolic alterations, particularly those related to oxidative stress, lipid metabolism, and energy pathways, may complement proteomic findings in reflecting tissue injury induced by chronic eye rubbing. Together, these multi-omics insights highlight a possible mechanistic link between allergic inflammation, mechanical trauma, and the development of LWE. However, further studies involving participants with LWE and a history of eye rubbing are required to confirm any direct relationship with AC and LWE from a multi-omics standpoint.

## 9. Clinical and Therapeutic Implications for Lid Wiper Epitheliopathy in Allergic Conjunctivitis

The potential responsiveness of LWE to treatment is clinically relevant because improvement in LWE could provide an indicator of improvement in lid wiper–ocular surface interaction. Although limited, there is evidence that perfluorohexyloctane, a non-aqueous ocular surface lubricant, was effective at treating LWE and improving symptoms in DED patients [123]. This finding provides clinical support for a potential role of tear film evaporation and ocular surface friction in LWE. If AC-associated LWE shares similar friction-mediated mechanisms with LWE in DED, lubricant-based interventions may likely expedite resolution in AC too. 

If allergic inflammation is the dominant driver, then anti-allergy treatments should in theory demonstrate superior benefit. However, whether pharmacological treatment of AC can accelerate LWE resolution remains unconfirmed, as evidence demonstrating that first line treatment options for AC such as mast cell stabilisers or antihistamines accelerate LWE resolution in patients with AC is limited. There is some evidence that systemic oral antihistamines may even have a negative effect, as they have been found to increase symptoms of DED and reduce tear break up time [124]. On the other hand, topical anti-allergy agents with dual antihistamine and mast cell stabilising activity such as ketotifen fumarate and olopatadine may show more promise; a clinical trial discovered that topical ketotifen fumarate 0.025% and olopatadine 0.1% eye drops demonstrated decreased expression of cell adhesion molecules and inflammatory markers on the conjunctival surface cells in patients with seasonal AC, providing biological plausibility for a potential link between the reduction in allergic inflammation and improvement of LWE, although it should be noted that LWE itself was not assessed [125]. 

Thus, targeting both ocular surface mechanical friction and allergic inflammatory pathways may provide a complementary therapeutic strategy for LWE in AC patients. Prospective studies assessing changes in AC-associated LWE after pharmacological intervention alongside measures of symptoms, eye rubbing behaviour, tear film parameters and ocular surface signs are required to determine whether LWE is a viable treatment-responsive endpoint and whether changes in LWE correspond with clinically meaningful improvement of AC overall.

## 10. Conclusions

Although LWE has been a well-established clinical sign of DED, it has not traditionally been associated with AC. However, recent evidence suggests that AC should also be considered as an important friction-mediated ocular surface disorder, especially when associated with eye rubbing. This review synthesises behavioural, mechanical and biophysical evidence to propose LWE as a plausible yet under-recognised manifestation of IgE-mediated AC. Although direct causal evidence remains limited, current literature consistently demonstrates that AC is characterised by ocular itch, frequent eye rubbing, tear film instability, reduced goblet cell function, altered mucin expression, and epithelial barrier dysfunction. Individually, each of these factors have been associated with increased ocular surface friction or epithelial injury. Collectively, they provide a biologically plausible mechanism through which AC may likely predispose to LWE.

Rather than acting through a single pathway, the available evidence suggests that LWE in AC is multifactorial. Tear film instability likely reduces lubrication during normal blinking, while ocular itch drives frequent and forceful eye rubbing, imposing significant mechanical load and friction on the lid wiper that is histologically specialised in its function, mucin-dependent for surface lubrication and intrinsically vulnerable to frictional stress. AC shares tear film deficiencies with DED, including reduced tear volume, tear instability, altered mucin organization and glycocalyx dysfunction, each of which may compromise ocular surface lubrication and increase susceptibility to friction-induced LWE. Together, these inflammatory, mechanical and lubricative insults form a coherent mechanistic framework that link AC to LWE. Concurrent inflammatory and metabolomic changes may further impair epithelial repair, mucin production and barrier integrity, perpetuating a cycle of friction, ocular surface damage and ocular discomfort. Furthermore, emerging proteomic, lipidomic and metabolomic studies provide important biological support for this hypothesis by identifying molecular signatures consistent with inflammation, oxidative stress, epithelial remodelling and wound healing in allergic ocular surface disease.

Despite these advances, significant gaps in the literature still remain. No studies have directly quantified ocular surface friction in AC, and the specialised biology of the lid wiper remains incompletely characterised, especially in younger individuals with AC. Similarly, direct investigation of eye rubbing-induced friction and LWE in AC is absent. Further studies should investigate objective friction measurement and behavioural monitoring of eye rubbing, lid wiper-specific mucin characterisation along with multi-omics approaches targeted to better define the biomechanical and molecular pathways that underpin LWE in patients with AC. Longitudinal studies evaluating whether treatment of allergic inflammation and reduction in eye rubbing improve LWE would also help establish causality and determine its potential as a therapeutic endpoint.

Overall, current literature supports the notion that LWE represents a clinically-accessible potential adjunctive clinical sign of cumulative frictional stress in AC. As understanding of ocular surface tribology continues to evolve, recognising the interaction between inflammation, tear film lubrication and mechanical forces may provide new opportunities for earlier diagnosis and targeted intervention in allergic eye disease.

## Figures and Tables

**Table 1:** Tear break-up times in allergic conjunctivitis participants across various studies as compared to normal controls. FBUT = fluorescein tear break-up time; NITBUT = non-invasive tear break-up time; AC = allergic conjunctivitis; C = control.

Study	Participant Age (years; Mean ± SD)	Technique	Allergic Conjunctivitis (seconds)	Control (seconds)	Sample Size (AC/Control)	*p*-Value
Polat & Tuncer (2014) [66]	AC = 28.8 ± 6.4, C = 25.9 ± 5.9	FBUT	12.76 ± 5.30	23.50 ± 4.45	26/20	0.001
Dogru et al. (2015) [65]	AC = 8.4 ± 2.8, C = 8.1 ± 2.5	FBUT	15.7 ± 4.4	18.4 ± 2.9	45/45	0.001
Suzuki et al. (2006) [67]	AC = 32.6, C = 32.5	FBUT	3.9 ± 2.1	12.4 ± 2.4	78/40	<0.05
Wei et al. (2024) [64]	AC = 11.78 ± 6.77, C = 11.65 ± 9	NITBUT	8.65 ± 6.31	10.48 ± 2.58	252/200	<0.05

**Table 2:** Multi-omics biomarkers implicated in AC-associated ocular surface change and proposed relevance to LWE.

Biomarker	Type	Direction	Primary Biopathway(s)	Proposed Role in LWE	References
AMY1A/B/C	Protein	Upregulated	Immune response	Stimulated in response to epithelial stress and wound healing response	[75]
KRT76 and FMOD	Protein	Upregulated	Wound healing, angiogenesis	Epithelial remodelling and wound healing response to mechanical stress	[75]
DKK4	Protein	Upregulated	Wound healing	Inhibition of Wnt signalling, resulting in tear film instability and disruption of ocular surface homeostasis under persistent mechanical stress and through reduced meibomian gland function	[75,107]
KNG1	Protein	Downregulated	Wound healing	Regulation of inflammatory cascades and vascular permeability	[75,119,120]
MSN	Protein	Downregulated	Cytoskeletal reorganisation, epithelial stability	Hypothesised to weaken cellular structural bonds under mechanical stress from eye rubbing	[75]
FLG	Protein	Downregulated	Epithelial barrier maintenance	Impaired epithelial barrier maintenance and increased vulnerability at the ocular surface and lid wiper region	[100]
KRT80	Protein	Downregulated	Epithelial differentiation, barrier integrity	Compromised barrier function and structural stability at mechanically stressed sites	[100]
CAPZA 1/2	Protein	Downregulated	Cytoskeletal integrity	Indicates loss of cellular integrity under mechanical stress from eye rubbing	[75,106]
miR-143-3p	miRNA	Downregulated	Wound healing	Implicated in inflammation, cellular repair and angiogenesis	[81,109]
miR-205-5p, miR-126-3p, and miR-516b-5p	miRNA	Dysregulated	Wound healing, angiogenesis, inflammation	Regulation of epithelial repair, vascular endothelial growth factor expression and inflammatory mediator expression. Dysregulation delays wound healing and promotes angiogenesis, contributing to further ocular surface damage	[81,110]
miR-133b	miRNA	Downregulated	Inflammation	Downregulation results in increased inflammatory mediators, promoting the allergic inflammatory cascade	[111,112,113]
Choline-derived lipids	Lipid	Downregulated	Tear film stability	Reduced tear film stability and increased evaporation, contributing to increased friction between the lid wiper and ocular surface	[114,115]
Phosphatidylcholine and Phosphatidylethanolamine	Lipid	Downregulated	Tear film integrity	Altered lipid layer composition contributing to inflammation, contributing to increased friction between the lid wiper and ocular surface	[114,115]
IL-1β, IL-18	Cytokine	Upregulated	Innate immune activation, pro-inflammatory signalling	Primary pro-inflammatory response to mechanical stress, initiates inflammatory cascade, amplifies epithelial dysfunction and barrier loss	[102,116,121,122]
IL-17A	Cytokine	Upregulated	Th17-mediated inflammatory amplification	Secondary amplification of the inflammatory cascade, drives epithelial dysfunction characterised by goblet cell apoptosis, mucin deficiency, and compromised barrier integrity	[102,117]
IFN-γ	Cytokine	Upregulated	Th1-mediated inflammatory amplification	Contributes to compromised barrier integrity and sustained LWE chronicity	[102,118]
PTEN and GG-NER	Metabolic pathway	Upregulated	Oxidative stress, DNA damage response	Hypothesised to reflect epithelial wound healing, although pathway activity was not directly measured and LWE was not assessed	[114]
Homogentisic acid, Hexanoylglycine, and Ornithine	Metabolite	Downregulated	Amino acid and lipid metabolism	Reduced metabolite components suggest compromised tear film stability and increased evaporation, enhancing friction between lid wiper and ocular surface	[114]

## Data Availability

No new data were created or analysed in this study. Data sharing is not applicable to this article.

## Abbreviations

The following abbreviations are used in this manuscript:

AC	Allergic conjunctivitis
AMY1A/B/C	Alpha-amylase 1A, 1B and 1C
CAPZA 1/2	F-actin capping protein subunit alpha
DED	Dry eye disease
DKK4	Dickkopf-related protein 4
DNA	Deoxyribonucleic acid
FBUT	Fluorescein break-up time
FLG	Filaggrin
FMOD	Fibromodulin
GG-NER	Global genome nucleotide excision repair
IgE	Immunoglobulin E
KNG1	Kininogen-1
KRT76	Keratin 76
KRT80	Keratin 80
LWE	Lid wiper epitheliopathy
MAM	Membrane-associated mucin
miRNA	Micro-ribonucleic acid
MSN	Moesin
NITBUT	Non-invasive tear break-up time
OA	Ocular allergy
OSD	Ocular surface disease
PTEN	Phosphatase and tensin homolog
SANDE	Symptom assessment in dry eye
TFOS DEWS III	Tear Film and Ocular Surface Society Dry Eye Workshop III
TMH	Tear meniscus height
